# Supplementary material for: The Characteristics and Obstetric Outcomes of Type II Vasa Previa: Systematic Review and Meta-Analysis
Source: Biomedicines. 2022 Dec 15;10(12):3263. doi: 10.3390/biomedicines10123263 (PMC9776262; doi:10.3390/biomedicines10123263)
Supplement: Supplementary file 1 [file biomedicines-10-03263-s001.zip › biomedicines-2095934-supplementary.pdf]

Supplemental File S1. The search strategy.

PubMed

#1 “abnormal placenta” [TIAB] OR “placenta abnormality” [TIAB] OR “placental variants” [TIAB]

#2 bilobate placenta [TIAB] OR bilobed placenta [TIAB] OR placenta bilobate [TIAB] OR bipartite placenta [TIAB] OR placenta duplex [TIAB] OR succenturiate placenta [TIAB] OR accessory lobe [TIAB] OR succenturiate lobe [TIAB]OR circumvallate placenta [TIAB] OR circummarginate placenta [TIAB] OR fenestrated placenta [TIAB] OR ring-shaped placenta [TIAB] OR zonary placenta [TIAB] OR placenta membranacea

#3 #1 OR #2

#4 Pregnancy outcome [MeSH] OR Pregnancy Complications [MeSH]

#5 “obstetrical complication” [TIAB] OR “maternal outcome” [TIAB] OR “fetal outcome” [TIAB] OR “neonatal outcome” [TIAB] OR “perinatal outcome” [TIAB] OR “maternal outcome” [TIAB] OR “surgical outcome” [TIAB] OR “pregnancy outcome” [TIAB]

#6 Placenta previa [MeSH] OR “placenta previa" [TIAB] OR "low lying placenta" [TIAB] OR "abnormal placenta*" [TIAB]

#7 Pre-Eclampsia [MeSH] OR Preeclampsia [TIAB] OR hypertension [TIAB] OR Fetal growth retardation [MeSH] OR “fetal growth restriction” [TIAB] OR “intrauterine growth restriction” [TIAB] OR “intrauterine growth retardation” [TIAB] OR Pre-Eclampsia [MeSH] OR preeclampsia [TIAB] OR “pre-eclampsia” [TIAB]

#8 Postpartum Hemorrhage [MeSH] OR “postpartum hemorrhage” [TIAB] OR Obstetric Labor Complications [MeSH] OR “obstetric labor complications” [TIAB] OR “labor complications” [TIAB] OR Abruptio Placentae [MeSH] OR “abruptio placentae” [TIAB] OR “placental abruption” [TIAB] OR “placental abruptions” [TIAB] OR Uterine Rupture [MeSH] OR “uterine rupture” [TIAB] OR “uterine ruptures” [TIAB]

#9 Dystocia [MeSH] OR “dystocia” [TIAB] OR “dystocias” [TIAB] OR Breech Presentation [MeSH] OR “breech presentation” [TIAB] OR “breech fetal presentation” [TIAB] OR “fetal malpresentation” [TIAB]

#10 Cesarean section [MeSH] OR cesarean section [TIAB] OR Cesarean delivery [TIAB] OR abdominal delivery [TIAB] OR “C section” [TIAB]

#11 Placenta accreta [MeSH]

#12 “Morbidly adherent placenta" [TIAB] OR "Morbid adherent placenta" [TIAB] OR “Placenta accreta spectrum" [TIAB] OR “Placenta accreta” [TIAB] OR "Placenta increta" [TIAB] OR "placenta percreta" [TIAB] OR "adherence of the placenta" [TIAB] OR "adherent placenta" [TIAB]

#13 “In Vitro Fertilization” [TIAB] OR fertilization in vitro [MeSH] OR Assisted Reproductive Techniques [MeSH] OR Embryo Transfer [MeSH] OR Intracytoplasmic Sperm Injection [MeSH] OR Cryopreserved [TIAB] OR "Oocyte donation" [TIAB] OR "fresh cycle" [TIAB] OR "frozen cycle" [TIAB] OR cleavage [TIAB] OR blastocyst [TIAB]

#14 #4 OR #5 OR #6 OR #7 OR #8 OR #9 OR #10 OR #11 OR #12 OR #13

#15 #3 AND #14

#16 “vasa previa” [TIAB]

#17 #15 OR #16

Cochrane Library
#1 “abnormal placenta”: ab,ti,kw OR “placenta abnormality”: ab,ti,kw OR “placental variants”: ab,ti,kw
#2 placenta bilobate: ab,ti,kw OR bipartite placenta: ab,ti,kw OR placenta duplex: ab,ti,kw OR succenturiate lobe: ab,ti,kw OR fenestrated placenta: ab,ti,kw OR ring-shaped placenta: ab,ti,kw OR zonary placenta: ab,ti,kw OR placenta membranacea
#3 bilobate placenta: ab,ti,kw OR bilobed placenta: ab,ti,kw OR succenturiate placenta: ab,ti,kw OR accessory lobe: ab,ti,kw OR succenturiate lobe: ab,ti,kw OR circumvallate placenta: ab,ti,kw OR circummarginate placenta: ab,ti,kw OR fenestrated placenta: ab,ti,kw
#4 #1 OR #2 OR #3
#5 Pregnancy outcome [MeSH]
#6 Pregnancy Complications [MeSH]
#7 “obstetrical complication”: ab,ti,kw OR “maternal outcome”: ab,ti,kw OR “fetal outcome”: ab,ti,kw OR “neonatal outcome”: ab,ti,kw OR “perinatal outcome”: ab,ti,kw OR “maternal outcome”: ab,ti,kw OR “surgical outcome”: ab,ti,kw OR “pregnancy outcome”: ab,ti,kw
#8 Placenta previa [MeSH]
#9 “placenta previa": ab,ti,kw OR "low lying placenta": ab,ti,kw OR "abnormal placenta*": ab,ti,kw
#10 Pre-Eclampsia [MeSH]
#11 Preeclampsia: ab,ti,kw OR hypertension: ab,ti,kw
#12 Fetal growth retardation [MeSH]
#13 “fetal growth restriction”: ab,ti,kw OR “intrauterine growth restriction”: ab,ti,kw OR “intrauterine growth retardation”: ab,ti,kw OR preeclampsia: ab,ti,kw OR “pre-eclampsia”: ab,ti,kw
#14 Postpartum Hemorrhage [MeSH]
#15 Obstetric Labor Complications [MeSH]
#16 “obstetric labor complications”: ab,ti,kw OR “labor complications”: ab,ti,kw
#17 Abruptio Placentae [MeSH]
#18 “abruptio placentae”: ab,ti,kw OR “placental abruption”: ab,ti,kw OR “placental abruptions”: ab,ti,kw
#19 Uterine Rupture [MeSH]
#20 “uterine rupture”: ab,ti,kw OR “uterine ruptures”: ab,ti,kw
#21 Dystocia [MeSH]
#22 “dystocia”: ab,ti,kw OR “dystocias”: ab,ti,kw
#23 Breech Presentation [MeSH]
#24 “breech presentation”: ab,ti,kw OR “breech fetal presentation”: ab,ti,kw
#25 Cesarean section [MeSH]
#26 Cesarean section: ab,ti,kw OR Cesarean delivery: ab,ti,kw OR abdominal delivery: ab,ti,kw OR “C section”: ab,ti,kw
#27 Placenta accreta [MeSH]
#28 “Morbidly adherent placenta": ab,ti,kw OR "Morbid adherent placenta": ab,ti,kw OR “Placenta accreta spectrum": ab,ti,kw OR “Placenta accreta”: ab,ti,kw OR "Placenta increta": ab,ti,kw OR "placenta percreta": ab,ti,kw OR "adherence of the placenta": ab,ti,kw OR

"adherent placenta": ab,ti,kw
#29 #5 OR #6 OR #7 OR #8 OR #9 OR #10 OR #11 OR #12 OR #13 OR #14 OR #15 OR #16 OR #17 OR #18 OR #19 OR #20 OR #21 OR #22 OR #23 OR #24 OR #25 OR #26 OR #27 OR #28
#30 #4 AND #29
#31 "Vasa previa": ab,ti,kw
#32 #30 OR #31

Scopus
#1 TITLE-ABS-KEY ("abnormal placenta" OR "placenta abnormality" OR "placental variants")
#2 TITLE-ABS-KEY ("abnormal placenta" OR "placenta abnormality" OR "bilobate placenta" OR "bilobed placenta" OR "succenturiate placenta" OR "accessory lobe" OR "succenturiate lobe" OR "circumvallate placenta" OR "circummarginate placenta" OR fenestrated placenta)
#3 TITLE-ABS-KEY ("placenta bilobate" OR "bipartite placenta" OR "placenta duplex" OR "succenturiate lobe" OR "fenestrated placenta" OR "ring-shaped placenta" OR "zonary placenta" OR "placenta membranacea")
#4 #1 OR #2 OR #3
#5 TITLE-ABS-KEY (pregnancy OR pregnanc* OR pregnant OR gravid* OR obstet* OR postpartum* OR birth OR fetus* OR foetus* OR fetal OR foetal OR gestation OR gestations OR "obstetrical complication" OR "maternal outcome" OR "fetal outcome" OR "neonatal outcome" OR "perinatal outcome" OR "maternal outcome" OR "surgical outcome" OR "pregnancy outcome")
#6 TITLE-ABS-KEY ("placenta previa" OR "low lying placenta" OR "abnormal placentation" OR "Morbidly adherent placenta" OR "Morbid adherent placenta" OR "Placenta accreta" OR "Placenta increta" OR "Placenta percreta" OR "adherence of placenta" OR "adherence of the placenta" OR "adherent placenta")
#7 TITLE-ABS-KEY (Preeclampsia OR "pre-eclampsia" OR hypertension OR "Fetal growth retardation" OR "fetal growth restriction" OR "intrauterine growth restriction" OR "intrauterine growth retardation" OR "placenta previa" OR "low lying placenta" OR "fetal growth restriction" OR "intrauterine growth restriction" OR "intrauterine growth retardation" OR preeclampsia OR "pre-eclampsia" OR "postpartum hemorrhage" OR "obstetric labor complications" OR "labor complications" OR "abruptio placentae" OR "placental abruption" OR "placental abruptions")
#8 TITLE-ABS-KEY ("uterine rupture" OR "uterine ruptures" OR "dystocia" OR "dystocias" OR "breech presentation" OR "breech fetal presentation" OR "fetal malpresentation")
#9 TITLE-ABS-KEY ("placenta accreta" OR "placenta accreta spectrum" OR "placenta increta" OR "placenta percreta")
#10 TITLE-ABS-KEY ("cesarean section" OR "cesarean delivery" OR "abdominal delivery" OR "C section")
#11 #5 OR #6 OR #7 OR #8 OR #9 OR #10
#12 #4 AND #11
#13 TITLE-ABS-KEY ("vasa previa")
#14 #12 OR #13

Supplemental Table S1. The definition of heterogeneity.

I^2 value	Heterogeneity	Analysis
0%–<30%	Low	Fixed-effect
30%–60%	Moderate	Random-effect
50%–90%	Substantial	Random-effect
75%–100%	Considerable	Random-effect

According to the *Cochrane Handbook for Systematic Reviews of Interventions* (ver 6.3), heterogeneity was determined per the I^2 value [1].

Supplemental Table S2. Risk of bias assessment for the comparator study.

Authors	Confounding	Selection	Classification of intervention	Deviations from interventions	Missing data	Measurement of outcomes	Reported results	Overall bias
Tachibana D[2]	🟤	🟤	🟣	⚫	⚫	🟣	🟣	🟤
Gross A[3]	🟤	🟣	🟣	⚫	⚫	🟣	🟣	🟣
Sutera M[4]	🟤	🟤	🟣	⚫	⚫	🟣	🟣	🟤
Liu N[5]	🟤	🟣	🟣	⚫	⚫	🟣	🟣	🟣
Westcott JM[6]	🟤	🟤	🟣	⚫	🟣	🟣	🟣	🟣
Melcer Y[7]	🟤	🟣	🟣	⚫	⚫	🟣	🟣	🟤

Risk of bias assessment was performed using the Risk Of Bias In Non-randomized Studies–of Interventions tool (ROBINS-I) [8-10].

🟠 Low risk of bias (the study is comparable to a well-performed randomized trial with regard to this domain)

🟣 Moderate risk of bias (the study is sound for a non-randomized study with regard to this domain but cannot be considered comparable to a well-performed randomized trial)

🟤 Serious risk of bias (the study has some important problems in this domain)

🔴 Critical risk of bias (the study is too problematic in this domain to provide any useful evidence on the effects of intervention.

⚫ No information on how to base a judgment on the risk of bias for this domain.

References

[1] Higgins JPT, Thomas J, Chandler J, Cumpston M, Li T, Page MJ, Welch VA (editors). Cochrane Handbook for Systematic Reviews of Interventions version 6.3 (updated February 2022). Cochrane, 2022. Available from www.training.cochrane.org/handbook. (accessed 09/30/2022).

[2] Tachibana D, Misugi T, Pooh RK, Kitada K, Kurihara Y, Tahara M, et al. Placental Types and Effective Perinatal Management of Vasa Previa: Lessons from 55 Cases in a Single Institution. Diagnostics (Basel, Switzerland). 2021;11.

[3] Gross A, Markota Ajd B, Specht C, Scheier M. Systematic screening for vasa previa at the 20-week anomaly scan. Acta obstetricia et gynecologica Scandinavica. 2021;100:1694-9.

[4] Sutera M, Garofalo A, Pilloni E, Parisi S, Alemanno MG, Menato G, et al. Vasa previa: when antenatal diagnosis can change fetal prognosis. Journal of perinatal medicine. 2021;49:915-22.

[5] Liu N, Hu Q, Liao H, Wang X, Yu H. Vasa previa: Perinatal outcomes in singleton and multiple pregnancies. Bioscience trends. 2021;15:118-25.

[6] Westcott JM, Simpson S, Chasen S, Vieira L, Stone J, Doulaveris G, et al. Prenatally diagnosed vasa previa: association with adverse obstetrical and neonatal outcomes. American journal of obstetrics & gynecology MFM. 2020;2:100206.

[7] Melcer Y, Maymon R, Pekar-Zlotin M, Levinsohn-Tavor O, Tovbin J, Jauniaux E. Evaluation of the impact of vasa previa on feto-placental hormonal synthesis and fetal growth. European journal of obstetrics, gynecology, and reproductive biology. 2017;215:193-6.

[8] Sterne JA, Hernan MA, Reeves BC, Savovic J, Berkman ND, Viswanathan M, et al. ROBINS-I: a tool for assessing risk of bias in non-randomised studies of interventions. BMJ. 2016;355:i4919.

[9] Danna SM, Graham E, Burns RJ, Deschenes SS, Schmitz N. Association between Depressive Symptoms and Cognitive Function in Persons with Diabetes Mellitus: A Systematic Review. PLoS One. 2016;11:e0160809.

[10] ROBINS-I detailed guidance (2016). https://www.riskofbias.info/welcome/home/current-version-of-robins-i/robins-i-detailed-guidance-2016. (accessed 09/30/2022).
